# Comparative short-term effectiveness of non-surgical treatments for insertional Achilles tendinopathy: a systematic review and network meta-analysis

**DOI:** 10.1186/s12891-023-06170-x

**Published:** 2023-02-07

**Authors:** Violet Man-Chi Ko, Mingde Cao, Jihong Qiu, Isaac Chun-Kit Fong, Sai-Chuen Fu, Patrick Shu-Hang Yung, Samuel Ka-Kin Ling

**Affiliations:** Department of Orthopaedics and Traumatology, Faculty of Medicine, The Chinese University of Hong Kong (CUHK), Hong Kong SAR, China

**Keywords:** Insertional Achilles tendinopathy, Non-surgical treatments, Physiotherapy, Rehabilitation

## Abstract

**Background:**

The incidence of Achilles tendinopathy has risen over the past decades. Insertional Achilles tendinopathy is characterised by tissue degeneration of the Achilles tendon from its insertion in the calcaneus to up to 2 cm proximally. This clinical condition is accompanied by pain, loss of function and diminished exercise tolerance. Numerous conservative treatment modalities are available to participants with insertional Achilles tendinopathy, including eccentric exercises, extracorporeal shockwave therapy, laser therapy, cryotherapy, therapeutic ultrasound, and orthotics. Eccentric exercise and extracorporeal shockwave therapy may reduce pain in participants with non-calcified insertional Achilles tendinopathy. However, no specific treatment is recommended over another due to the low methodological quality of trials. Given the lack of standard or preferred non-surgical treatment and the potential risks of surgical treatment, there is an imminent need to reassess different non-surgical treatments based on the newest evidence. Thus, this systematic review aims to evaluate the clinical effectiveness of the various non-surgical treatments for insertional Achilles tendinopathy.

**Methods:**

AMED EBSCOhost, CINAHL, EBSCOhost, EMBASE, PEDro, PubMed, Web of Science, and Clinicaltrials.gov were searched from 1992 to 14th October 2022, randomised controlled trials of adults with insertional Achilles tendinopathy investigating non-surgical treatments compared with each other or no treatment, placebo/sham control. Two reviewers independently screened and extracted the data. Random effects of network meta-analysis immediately after treatments were used to report comparative treatment effects. The surface under the cumulative ranking probabilities was calculated to assess the relative ranking of treatments.

**Results:**

Nine trials (total *n* = 464 participants) were included. This review recommended the combination of eccentric exercise and soft tissue therapy to manage insertional Achilles tendinopathy. With the highest SUCRA values of 84.8, and the best mean rank of 1.9, Eccentric exercise plus soft tissue treatment ranked as the most effective treatment for short-term pain.

**Conclusions:**

This is the first NMA of non-surgical treatment focusing on short-term pain control for IAT which eccentric exercise plus soft-tissue therapy was found to be the most effective treatment combination. However, the overall confidence in non-surgical treatments from all included trials was very low. No recommendation of the best treatment option can be made from this review.

## Introduction

The incidence of Achilles tendinopathy (AT) has risen over the past decades [1]. The overall incidence rate of Achilles tendinopathy was 2.35 per 1000 in adults between 21 and 60 years [2]. Almost 6% of the general population will suffer from AT in a lifetime [3]. In the past decades, much effort has been made to understand the pathophysiology of AT. Histopathological studies have shown that AT is characterised by progressive degeneration of the tendon tissue and poor natural healing [4].

To explain the condition’s progression, previous studies described tendinopathy as a three-stage process: injury of collagen fibrils, inadequate healing followed by symptom manifestation [5]. Various theories have been proposed to explain how collagen fibrils are initially injured, including but not limited to mechanical stress, inflammation, apoptosis, and tendon weakening due to increased vascular ingrowth [5]. Following such an injury, the healing response will be launched. Nevertheless, there is inadequate or failed tendon healing under various extrinsic and intrinsic factors, though this is still asymptomatic [5, 6]. Without adequate healing, symptoms start to appear due to the accumulation of microstructural damage, associated mediators and activation of nociceptors [5]. In AT, these symptoms are typically pain, stiffness and swelling in the region [7, 8].

Clain and Baxter divided Achilles tendon disorders into midportion and insertional tendinopathy in 1992 [9]. Since then, various studies have described them as two entities that are clinically distinct from each other [9, 10]. IAT is characterised by tissue degeneration of the Achilles tendon from its insertion in the calcaneus to up to 2 cm proximally [11]. IAT involves the tendon-bone interface and may be associated with a prominent posterosuperior calcaneal tuberosity (Haglund’s deformity). IAT’s primary symptoms were reported as early morning stiffness and pain localised at Achilles tendon insertion that worsens with activity. Physical examination typically shows tenderness and thickening at the insertion site and limited dorsiflexion [9]. Exercise rehabilitation and extracorporeal shockwave therapy (ESWT) were reported to be effective in treating IAT [10, 12].

MAT is tendon substance degeneration in the relatively hypovascular region of the Achilles tendon that is 2 to 6 cm proximal to the calcaneal insertion, the same as the typical site of Achilles tendon ruptures [13]. The primary symptom of MAT was pain on the tendon’s posteromedial side, which can interfere with function, especially during exercise. At the same time, the typical sign is tenderness and swelling over the symptomatic area [14]. As for the management of MAT, a recent systematic review suggested that there is currently a lack of high-quality evidence to justify any interventions for MAT, but calf muscle exercise could be used as an initial intervention due to its low cost and wide availability [15].

IAT and MAT are diagnosed based on the symptoms and signs obtained from history and physical examination [14]. Subsidiary exams such as ultrasound could help to assess the severity of the condition [16]. In contrast, X-rays could help to exclude differential diagnoses and to visualise Haglund’s deformity and calcifications which are often seen in cases of IAT [12].

There is a consensus, but limited evidence, that non-surgical methods should be attempted before surgery is advocated [17]. Although surgical treatment can be effective, it has surgical risks and requires substantial rehabilitation before returning to sport [18]. A large-scale clinical study of 432 participants illustrated an overall complication rate of 11%, with 3% requiring a reoperation. Most common were wound problems with a wound necrosis rate of 3%, a superficial infection rate of 2.5%, and a superficial nerve damage rate of 1% [19]. Participants are not recommended to resume competition until 6 months after surgery, similar to those with Achilles tendon repair after an acute rupture [20].

Numerous non-surgical treatment modalities are available to participants with IAT, including eccentric exercises, extracorporeal shockwave therapy, laser therapy, cryotherapy, therapeutic ultrasound and orthotics [21]. Although eccentric exercise and shockwave therapy have been reported to reduce pain in participants with non-calcified IAT [10], the results were based on limited studies. A previous network meta-analysis on MAT showed that calf muscle exercises could be used as an initial treatment [15]. However, no specific treatment is recommended over another due to the low methodological quality of trials.

In another systematic review, shockwave therapy combined with eccentric exercise was recommended in the MAT treatment in a previous systematic review [22]. More high-quality evidence is necessary to be more conclusive regarding the best available treatment [10]. Given the lack of standard or preferred non-surgical treatment and the potential risks of surgical treatment, there is an imminent need to reassess different non-surgical treatments based on the newest evidence. Thus, this systematic review aims to evaluate the clinical effectiveness of various non-surgical treatments for IAT.

## Methods

### Protocol and registration

This review was prospectively registered on PROSPERO (International Prospective Register of Systematic Reviews). The review protocol followed the Preferred Reporting Items for Systematic Reviews and Meta-Analyses (PRISMA) guideline.

### Search strategy and selection criteria

A systematic search of the literature was undertaken on multiple databases. The following databases were searched for published and unpublished trials from 1992 to 14^th^ October 2022: AMED EBSCOhost, CINAHL, EBSCOhost, EMBASE, PEDro, PubMed, Web of Science, and Clinicaltrials.gov. Boolean operators were used to maximising relevant results and minimise irrelevant results (Table 1). We examined the reference lists of identified papers for potentially eligible trials. The language of the article was restricted to English.

**Table 1:** Search strategy

Electronic database	Search words
AMED EBSCOhost (Total: 14)	insertional AND (Achilles or calcaneal) AND (tendinopathy or tendonitis or tendinosis or tendinitis) AND (conservative treatment or conservative management or non-surgical or nonoperative)
CINAHL EBSCOhost (Total: 423)	insertional AND (Achilles or calcaneal) AND (tendinopathy or tendonitis or tendinosis or tendinitis) AND (conservative treatment or conservative management or non-surgical or nonoperative)
EMBASE (Total: 118)	insertional AND (Achilles or calcaneal) AND (tendinopathy or tendonitis or tendinosis or tendinitis) AND (conservative treatment or conservative management or non-surgical or nonoperative)
PubMed (Total: 52)	insertional AND (Achilles or calcaneal) AND (tendinopathy or tendonitis or tendinosis or tendinitis) AND (conservative treatment or conservative management or non-surgical or nonoperative)
Web of Science (Total: 102)	insertional AND (Achilles or calcaneal) AND (tendinopathy or tendonitis or tendinosis or tendinitis) AND (conservative treatment or conservative management or non-surgical or nonoperative)
Clinicaltrials.gov (Total: 13)	insertional Achilles tendinopathy

### Eligibility criteria and study selection

Studies eligible for this review were randomised controlled trials of participants with IAT comparing a non-surgical treatment with other non-surgical treatments, with no intervention or placebo control. IAT must have been diagnosed based on clinical findings, while imaging was not a pre-requisite. Trials including both athletes and normal populations were eligible.

Studies on the treatment for MAT or unspecified Achilles tendinopathies were excluded. Studies combining the results of MAT and IAT were also excluded if the results of IAT could not be analysed separately. All studies on pathology located at or around the Achilles tendon insertion but not diagnosed as IAT were excluded. Animal, in vitro studies, case series, and cohort studies were excluded. Non-surgical treatments were a variety of techniques conventionally used by doctors and physiotherapists to treat participants with IAT, such as active participation in treatment by the participants, hands-on techniques applied by the physiotherapists and medication prescription by doctors.

### Quality assessment and data extraction

Two review authors (VMCK and MC) independently read all articles. Two independent reviewers identified titles and abstracts after duplicate removal. Reviewers applied eligibility criteria to the full-text reports alone. These two reviewers identified all titles, assessed the abstracts independently and excluded any irrelevant articles. Reviewers discussed any remaining differences and resolved the disagreements by consensus.

The PEDro scale was chosen to rate the methodological quality of the included RCTs in terms of randomisation and blinding, concealment of allocation, data collection and analysis (Table 2). A total of 11 criteria were included in the PEDro scale, each satisfying criteria except criteria 1, which contributed one point to the total score. The reliability of the PEDro scale was reported to be sufficient for use in systematic reviews relevant to physiotherapy [31]. The assessment was performed independently by two reviewers. Disagreements were resolved via consensus or by a third reviewer (JQ) if necessary [32].

**Table 2:** Data extraction table

Author (year)	Study design	Age	Method of diagnosis	Follow-up	Bony disorder	Intervention 1	Intervention 2	Outcomes	Results
Kedia et al. (2014) [23]	RCT (*n* = 36)	54	Clinical examination	6, 12 weeks	Not reported	CPT	CPT, EE	VAS,SF-36, FAOQ, AROM, MMT	Significant improved VAS (E: 2.43 ± 1.99 vs C: 1.50 ± 2.16), SF-36 (E: 70.00 ± 19.95 vs C: 70.50 ± 19.97), FAOQ (E: 0.78 ± 0.58; C: 0.74 ± 0.75) in both groups. No significant differences between groups. VAS: *p* = 0.129, SF-36: *p* = 0.789, FAOQ: *p* = 0.464
Notarnicola et al. (2012) [24]	RCT (*n* = 64)	55.8	X-ray, US, MRI	10th-15th days, 8, 24 weeks	Not reported	Placebo ESWT	AS, ESWT	VAS, AOFAS AHS, RMS	Significant improved VAS at 8 weeks in both groups. No significant differences between groups. (E:3.9, 3.2 vs C:5.1, 2.7, *p* = 0.07)
Notarnicola et al. (2014) [25]	RCT (*n* = 60)	58.5	X-ray, US, MRI	10th-15th days, 8, 24 weeks	Not reported	CHELT	ESWT	VAS, AOFAS AHS, RMS	Significant improved VAS at 8 (*p* < 0.0001) and 24 weeks (*p* < 0.0001). VAS was lower in CHELT group at 8 (CHELT: 2.3, 1.1 vs ESWT: 4.9, 0.9) and 24 weeks (CHELT: 2.4, 1.6 vs ESWT: 5.4, 2.7).
Pinitkwamdee et al. (2020) [26]	RCT (*n* = 31)	59	Clinical examination, X-ray, US, MRI	2, 3, 4, 6, 12, and 24 weeks	Haglund deformity, Calcification	ESWT, CPT	Placebo ESWT	VAS, VAS-FA	No significant difference in VAS (6.0 ± 2.6 vs 5.2 ± 2.2) and VAS-FA (64.8 ± 16.6 vs 65.3 ± 12.7) between groups in follow-ups.
Mansur et al. (2021) [11]	RCT (*n* = 119)	52.9	Clinical examination, US	2, 4, 6, 12, and 24 weeks	Haglund deformity	ESWT + EE	EE	VAS, VISA-A, SF-12, algometry, FAOS	Significantly improved all outcomes. No between-group differences in any of the outcomes. (all *p* > 0.05)
Horstmann et al. (2013) [27]	RCT (*n* = 58)	46	Clinical examination, US	12 weeks	Not reported	WBV	EE, WS	VAS, Likert scale, muscle strength and flexibility	Significant improved VAS in all groups. No between-group differences in VAS (all p > 0.05).
Gatz, Matthias et al. (2020) [28]	RCT (*n* = 30)	48.4	Clinical examination, US	12 weeks	Not reported	EE	IS	VISA-A, AOFAS AHS, Likert scale, RMS	Significant improved VISA-A in all groups. No between-group differences in VISA-A (*p* = 0.362).
McCormack et al. (2016) [29]	RCT (*n* = 16)	53.6	Clinical examination	4, 8, 12, 26, and 52 weeks	Not reported	ST	EE	NPRS, VISA-A, GROC	Both groups experienced a similar statistically significant improvement in pain over the short and long term (*p* = 0.02). A significantly substantial number of subjects in the Astym group achieved a successful outcome at 12 weeks (*p* = 0.01).
Rompe et al. (2008) [30]	RCT (*n* = 50)	39.8	Clinical examination	16, 52 weeks	Not reported	EE	ESWT	NPRS, VISA-A, Likert scale	For all outcome measures, the group that received shock wave therapy showed significantly more favourable results than those treated with eccentric loading (*p* = 0.002).

*AHS* Ankle–Hindfoot Scale, *AOFAS* American Orthopaedic Foot & Ankle Society Ankle-Hindfoot Scale, *AROM* Ankle Range of Motion, *CHELT* Cryotherapy, high energy laser therapy, *CPT* Conventional physiotherapy (Stretches, ice massage, bilateral heel lifts, resting night splints), *EE* Eccentric calf muscle training, *ES* Effect size, *ESWT* Extracorporeal shock wave therapy, *FAOQ* Foot and Ankle Outcomes Questionnaire, *GROC* Global Rating of Change scale, *IS* Isometric calf muscle training, *MMT* Manual Muscle Test of the gastrocnemius, *NPRS* Numerical Pain Rating Scale, *RMS* Roles and Maudsley Score, *SF-36* Short Form Health Survey, *ST* soft tissue therapy, *VAS* Visual Analogue Scale, *WBV* Whole body vibration, *WS* Wait-and-see

The Grading of Recommendations, Assessment, Development and Evaluations (GRADE) was used to rate the overall quality of evidence and guide clinical recommendations based on the certainty of evidence (Table 3). The summary of findings will be presented for the short-term comparative effectiveness of non-surgical treatments for IAT.

**Table 3:** Pedro’s scores of each included study

Author (year)	Criteria for PEDRO scale ^a^										
	1	2	3	4	5	6	7	8	9	10	11
Kedia et al. (2014) [23]	√	√	√	√	x	√	x	√	√	√	√
Notarnicola et al. (2012) [24]	√	√	√	√	√	x	√	√	√	√	√
Notarnicola et al. (2014) [25]	√	√	√	√	x	x	√	√	√	√	√
Pinitkwamdee et al. (2020) [26]	√	√	√	√	√	x	√	√	√	√	√
Mansur et al. (2021) [11]	√	√	√	√	√	√	√	√	√	√	√
Horstmann et al. (2013) [27]	√	√	√	√	x	x	√	√	√	√	√
Gatz, Matthias et al. (2020) [28]	√	√	√	√	x	x	x	√	√	√	√
McCormack et al. (2016) [29]	√	√	√	√	x	x	x	√	√	√	√
Rompe et al. (2008) [30]	√	√	√	√	x	x	x	√	√	√	√

^a^1: eligibility criteria were specified; 2: subjects were randomly allocated to groups; 3: allocation was concealed; 4: the groups were similar at baseline regarding the most important prognostic indicators; 5: there was blinding of all subjects; 6: there was blinding of all therapists who administered the therapy; 7: there was blinding of all assessors who measured at least one key outcome; 8: measures of at least one key outcome were obtained from more than one of the subjects initially allocated to groups; 9: all subjects for whom outcome measures were available received the treatment or control condition as allocated or, where this was not the case, data for at least one key outcome was analysed by"intention to treat"; 10: the results of between-group statistical comparisons are reported for at least one key outcome; 11: the study provides both point measures and measures of variability for at least one key outcome

### Data analysis and synthesis

Results of trials reporting VAS and NPRS (0-10) were combined using network meta-analytic methods to estimate an overall effect for non-surgical treatment. In VAS and NPRS, 0 indicates “no pain”, and 10 means “very severe pain”. The level of pain was chosen to measure effectiveness using meta-analysis because a reduction in pain was always the treatment goal for clinicians and patients in a clinical setting. The VAS and NPRS reported in the included trials referred to the pain at the time after treatment was given to the participants. There appeared to be no evidence documenting the MDC and MCID of VAS and NPRS for IAT. The minimally clinical important difference (MCID) of NPRS was shown to vary between 1.7 points [33] and 2 points [34] for chronic musculoskeletal pain. The MCID of VAS was shown to range from 1.8 to 5.2 points after foot and ankle surgery. The minimal detectable change (MDC) of VAS (0-10) and NPRS was reported to be 2.8 cm [35] and 4.3 points [36] for participants with other chronic musculoskeletal pain.

**Fig. 1:**
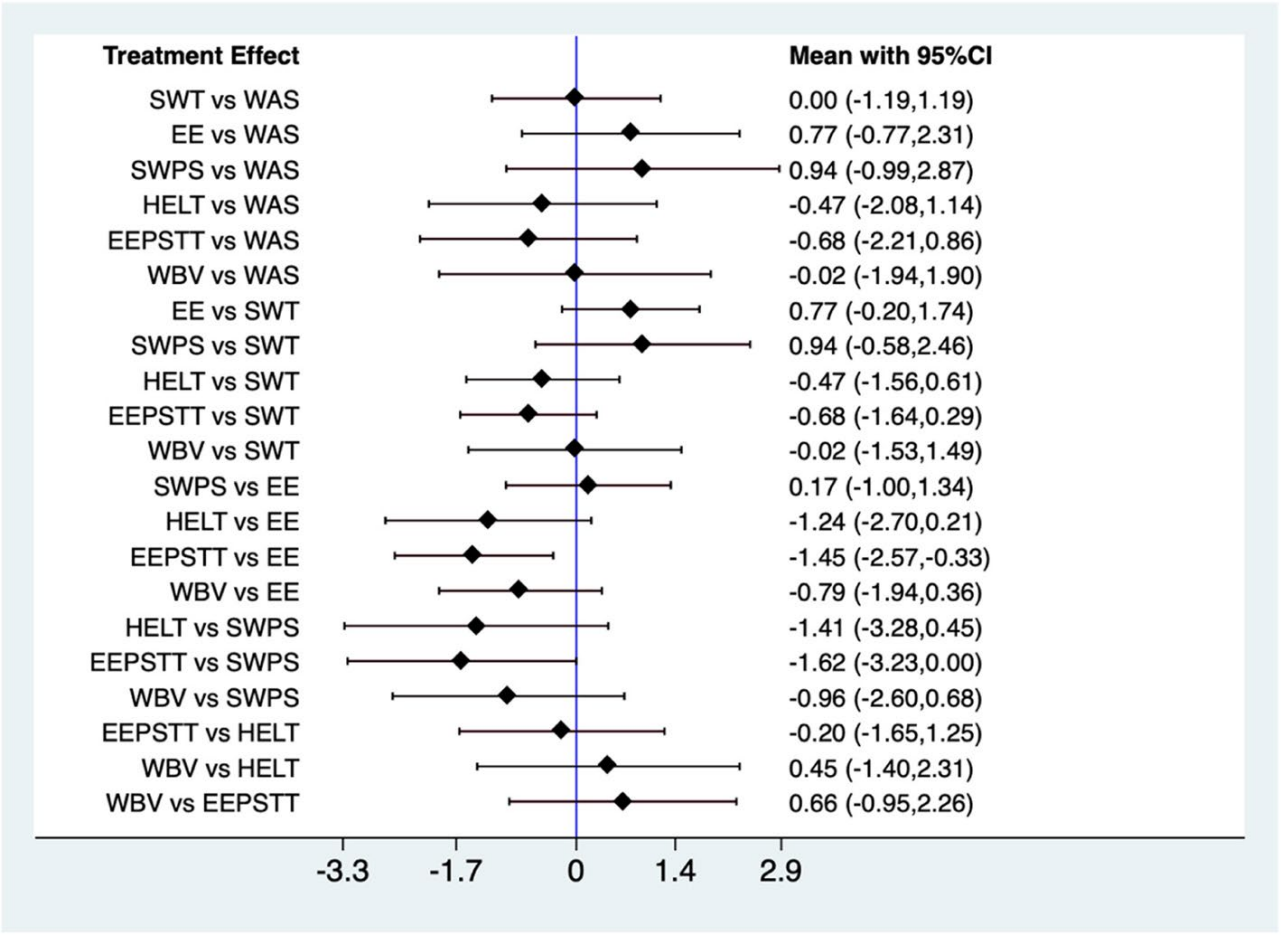
Forest plot. shockwave therapy, *EE* eccentric exercise, *SWPS* Shockwave therapy plus dietary supplementation, *HELT* high-energy laser therapy, *EEPSTT* eccentric exercise plus soft tissue therapy, *WBV* whole-body vibration, *WAS* wait-and-see approach

Effect sizes were considered small (d ≤ 0.2), medium (d ≤ 0.5), and large (d ≥ 0.8) (Fig. 1) [37]. Weighted mean difference methods were used as all outcomes were continuous variables: the mean change and standard deviation from baseline to the time point after the intervention was calculated.

The primary analysis compared non-surgical treatments using change in pain from baseline to the first assessment after treatment. It was immediately after the intervention in most trials. This comparison was chosen because it was the primary data analysis reported in most trials and few reported data at assessment points in the longer term.

To assess the comparative effectiveness of treatments, the ranking probability distributions of each treatment were generated from a simulation of 5000 replications (Table 4). Mean rank, surface under simulative ranking curve (SUCRA) values and cumulative ranking plots were used to show the comparative effectiveness of treatments. These statistics rank treatments according to their ability to generate the most significant treatment effects in each simulation and are averaged over the 5000 replications [38].

**Table 4:** Network meta-analysis treatment ranking results for pain immediately after the treatment period

Treatment	SUCRA	Mean rank
EE + ST ^a^	84.8	1.9
CHELT ^a^	74.1	2.6
WBV ^a^	55.6	3.7
Wait-and-see ^a^	52.7	3.8
ESWT ^a^	51.8	3.9
EE ^a^	16.1	6.0
ESWT + supplement ^a^	14.9	6.1

^a^*CHELT* cryotherapy high energy laser therapy, *EE* Eccentric exercise, *ESWT* Extracorporeal Shockwave therapy, supplement: dietary supplement, *ST* soft tissue therapy, Wait-and-see: wait-and-see approach, *WBV* whole-body vibration

**Fig. 2:**
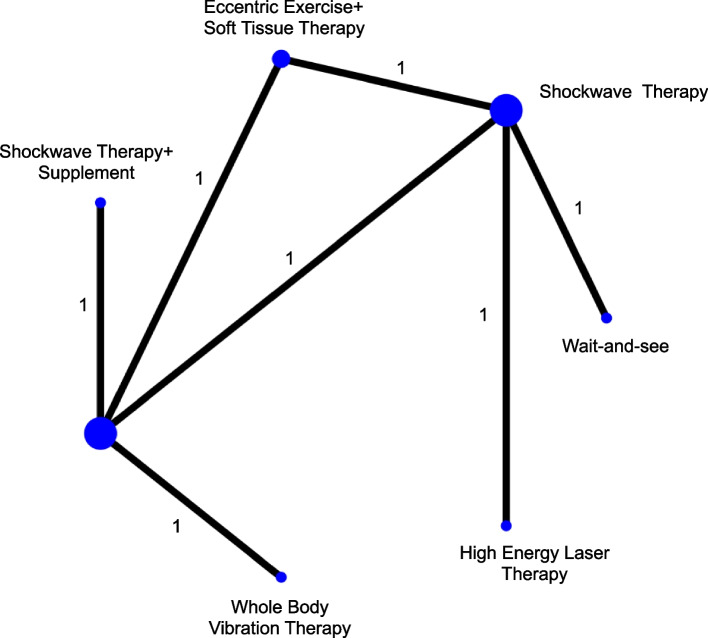
Network plots for treatment classes on the VISA-A score immediately in participants with insertional Achilles tendinopathy

A network graph consisting of “nodes” and “edges” was used to represent the non-surgical treatment and available comparisons between pairs of treatments (Fig. 2). When there was significant evidence, there was an increase in the nodes’ size and the line’s thickness. This graphical display was reported to guide the initial interpretation of the results for making clinical decisions.

## Results

**Fig. 3:**
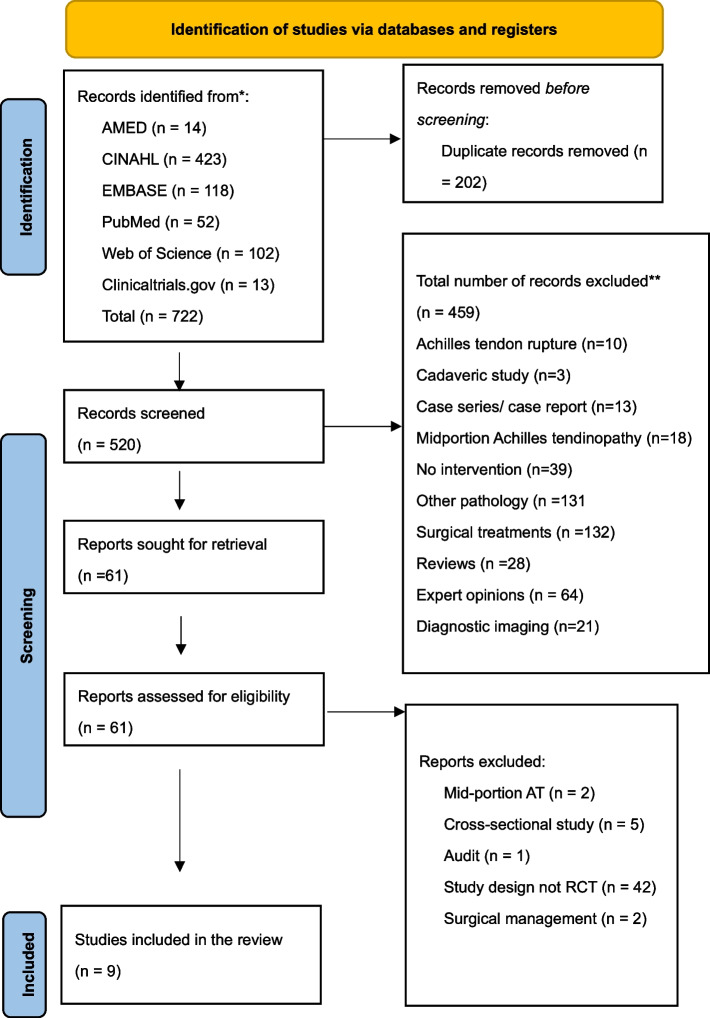
PRISMA flow diagram 2020 of the study selection process [39]

Figure 3 illustrates the process of study identification and selection. After combining the results and removing duplicates of these searches, 722 papers were screened based on title, abstract and study design. Four hundred fifty-nine articles were excluded. The full text of 61 papers was obtained and assessed for eligibility. The exclusion was based on the study design. Articles were excluded based on full text, including nine articles for this review.

### Characteristics of included studies

The total number of participants with IAT in the nine included studies was 464. Overall, the age of included trials ranged between 18 and 80 (one study did not report patient characteristics) (Table 2).

Of the included studies, the scores on the Pedro scale ranged from eight to ten, with a median score of nine and one study has fulfilled all criteria [40]. All studies have met most criteria except for the blinding of all subjects in six studies [23, 25, 27–30], blinding of therapists who administered the therapy in seven studies [24–30], and assessors who measured at least one key outcome in four studies [23, 28–30]. As expected, it is impossible to blind both the subjects and therapists and assessors in most trials due to the nature of the study protocol, such as giving comparison of different interventions.

### Programme designs

The heterogeneity of intervention delivered among different studies was enormous. Eight treatment options were investigated in nine trials, including conventional physical therapy, eccentric exercise, isometric exercise, extracorporeal shockwave therapy, cold air and high-energy laser therapy, arginine supplementation, whole-body vibration, soft tissue treatment, and wait-and-see [23–30, 40]. Eccentric exercise was used primarily in the trials, followed by extracorporeal shockwave therapy. The total duration of intervention ranged from three sessions to 12 weeks. The frequency of sessions ranged from two sessions per week to five sessions per day. Regarding the intervention components, four studies used a single treatment in the intervention [25, 27–30], whilst the other five applied a combination of treatments.

### Data available for analysis

Seven of the nine trials reported in the extraction table had VAS or NPRS data for network meta-analysis [23–27, 29, 30]. There were seven treatment comparisons in the network meta-analyses (Fig. 2).

### Individual treatment effects

#### Comparison of eccentric exercise with other intervention

Six trials evaluated the effects of conventional physiotherapy and eccentric exercise on IAT [23, 26–28, 40, 41]. Based on the study from Kedia et al., the control group performed gastrocnemius, soleus and hamstring stretch, ice massage on the Achilles tendon twice a day, bilateral heel lifts and a resting night splint [23]. The experimental group followed everything in the control group by adding two eccentric strengthening exercises [23]. Significant improvements in VAS were found in both groups of participants (Experimental group: 2.43 ± 1.99, control group: 1.50 ± 2.16). The improvements were clinically meaningful. No statistically significant differences were found in patient outcomes in the two groups (VAS: *p* = 0.129, SF-36: *p* = 0.789, FAOQ: *p* = 0.778). One participant required physical therapy for knee pain that occurred after eccentric training.

Gatz et al. evaluated the effects of isometric calf muscle training compared with eccentric exercise using the Victorian Institute of Sports Assessment – Achilles questionnaire (VISA-A) and shear wave elastography (SWE) [28]. The VISA-A improved significantly in both groups, but there were no significant between-group differences (VISA-A; *P* = 0.362) [28]. The authors reported that no additional clinical benefits of adding isometric exercise to a basic eccentric exercise program could be found in this RCT over a period of 3 months [28]. McCormack et al. studied the effects of soft tissue treatment and eccentric exercise for IAT [29]. More significant improvements in the VISA-A were noted in the soft tissue treatment group (*p* = 0.02) during the 12-week treatment period, and these differences were maintained at the 26- and 52-week follow-ups (*p* < 0.01) [29]. A similar statistically significant improvement in pain over the short and long term was shown in both groups. In short- and long-term follow-ups, soft tissue treatment plus eccentric exercise was more effective than eccentric exercise only at improving function [29].

#### Comparison of shockwave therapy with other interventions

Five studies tested the effect of shockwave therapy for IAT [24–26, 30, 40]. One of them was a double-blinded randomised controlled trial. Pinitkwamdee et al. compared a low-energy shockwave therapy group with sham controls. There was no significant improvement in VAS in the long term between the two groups. In addition, the shockwave therapy group significantly improved in VAS (2.9 ± 2.2) from weeks 4 to 12, and the control group significantly improved VAS (2.3 ± 2.6) from weeks 12 to 24. Complications were found only after shockwave treatment. Two participants felt pain during the intervention, and two other participants were scheduled for surgery.

Notarnicola et al. evaluated the effects of cold air and high-energy laser therapy versus shockwave therapy in the treatment of insertional Achilles tendinopathy [25]. One group of participants received laser therapy, while the other received shockwave treatment. Statistically significant improvement of the VAS immediately after treatment, 2 months and 6 months follow-up examinations. The difference between the two groups was statistically significant in favour of the laser therapy group (*p* < 0.001) [25].

Horstmann et al. investigated the effects of whole-body vibration in AT participants with symptoms in the insertion, midportion and musculotendinous junction [27]. Compared with other groups, the most significant improvement was in pain at the musculotendinous junction in the eccentric exercise group. Most participants improved in the whole-body vibration group, followed by the eccentric exercise group. A whole-body vibration group could be a treatment adjunct in participants who do not respond well to eccentric exercise, especially those with insertional pain [27].

Notarnicola et al. prospectively compared shockwave therapy and arginine supplementation for insertional Achilles tendinopathy [24]. The VAS score at 6 months was significantly lower in the experimental group compared to the control group (2.0 vs 2.9; *P* = 0.04).

Table 4 shows the comparative effectiveness of treatments from a network meta-analysis. Results are based on a simulation of 5000 replications. Higher SUCRAs and lower mean rank indicate better-performing treatments. With the highest SUCRA values of 84.8, and the best mean rank of 1.9, Eccentric exercise plus soft tissue treatment ranked as the most effective treatment for short-term pain [29]. In contrast, shockwave therapy plus arginine supplementation was the least effective for pain relief in the short term [24].

were randomly allocated an order in which treatments were received); 3: allocation was concealed; 4: the groups were similar at baseline regarding the most important prognostic indicators; 5: there was blinding of all subjects; 6: there was blinding of all therapists who administered the therapy; 7: there was blinding of all assessors who measured at least one key outcome; 8: measures of at least one key outcome were obtained from more than 85% of the subjects initially allocated to groups; 9: all subjects for whom outcome measures were available received the treatment or control condition as allocated or, where this was not the case, data for at least one key outcome was analysed by “intention to treat”; 10: the results of between-group statistical comparisons are reported for at least one key outcome; 11: the study provides both point measures and measures of variability for at least one key outcome.

## Discussion

This review combined evidence from the trials evaluating various non-surgical methods into one study to assess the overall effect. It also allowed an indirect comparison of the different non-surgical methods used.

The PEDro scale evaluated the quality of all included studies (Table 3). All included studies were RCTs which scored more than seven out of ten points in Pedro [23–27, 29, 30]. However, some publications did not thoroughly discuss several quality indicators, as stated in Pedro. A significant proportion is needed to report how randomisation was performed adequately. Only three studies blinded both assessors and participants to the assigned interventions [24, 26, 40]. Seven studies did not blind the therapists who administered the therapy. A high risk of bias was considered present most frequently concerning the lack of blinding of participants and assessors. Most trials did not report sufficient information to assess the concealment of treatment allocation accurately.

The overall certainty of evidence was assessed using the GRADE approach (Table 5). All outcomes apart from pain are rated low based on inconsistency primarily due to the differences in the location of symptoms on the Achilles tendon and disease stages. Including sub-acute and chronic IAT signifies that treatment response may differ. In terms of pain, the certainty of the evidence was rated down to very low as 63% of studies did not blind all subjects, 75% did not blind all therapists who administered the therapy, and 38% did not blind all assessors. Furthermore, inconsistency and indirectness were also detected in 25 and 13% of studies, respectively. These and the network meta-analysis results reflect that the overall confidence in no n-surgical treatment for improving pain is very low. Overall, findings regarding treatment effectiveness should be evaluated with caution.

**Table 5:** Summary of findings in treatment rankings (GRADE approach) for all outcomes in studies investigating the efficacy of non-surgical treatments for insertional Achilles tendinopathy

Outcome	Number of Participants (Studies)	Certainty of evidence	Comments
Attitudes	138 (3)	Low	Inconsistency^a^
Range of motion	94 (2)	Low	Inconsistency^a^
Muscle strength	94 (2)	Low	Inconsistency^a^
Pain	434 (8)	Very Low	Risk of bias^c^ Inconsistency^a^ Indirectness^d^
Quality of life	325 (6)	Low	Inconsistency^a, b^
Activity limitations	154 (2)	Low	Risk of bias^c^
Physical function	251 (5)	Low	Inconsistency ^b^

Certainty of the evidence: very low, low, moderate, high

^a^Heterogeneity in the location of symptoms (i.e., up to 6 cm proximal to the insertion)

^b^Heterogeneity in stages of diseases

^c^Serious risk of bias due to the lack of blinding of all subjects, blinding of all therapist who administers the therapy and blinding of all assessors

^d^Differences in therapist contact time between group

The moderate effect size was reported in six included trials for VAS and NPRS, as shown in Table 2 [23–27, 29, 30]. However, for the most part, improvements tended to be short-term. Eccentric exercise and shockwave therapy were commonly investigated, which shared the same results from previous systematic reviews on IAT [10]. Dietary supplement plus shockwave therapy was shown to induce better clinical outcomes and reduced tendon perfusion in participants with IAT. Visual Analogue Scale, the Ankle-Hindfoot Scale, and the Roles and Maudsley score were used as outcome measures. All these scores were collected before treatment and 2 and 6 months after treatment during the follow-up examinations. The tissue perfusion values of the treated tendon were measured by oximetry at baseline assessment, the second and third shockwave therapy session, and 2 and 6 months of follow-ups.

The NMA identified that most studies compared interventions with eccentric exercise or shockwave therapy. However, none of the treatment comparisons showed a significant difference. In the NMA of pain, shockwave therapy plus arginine supplementation and eccentric exercise alone were generally shown to be the least effective treatment options [24]. The effect of non-surgical treatments in reducing pain appeared to be more prominent among participants with MAT. One parallel prospective study performed on 53 participants with MAT reported that PEMF effectively reduced pain [42]. At the 12-week assessment point, the VAS in both groups had significantly decreased, although the active group had improved considerably in VAS (*p* < 0.00001) compared with the controls (*p* = 0.0002). Nevertheless, the methodological quality of this study was limited as participants were not blinded to treatments [42].

This review provides evidence of the short-term effectiveness of non-surgical treatments in the short term in treating IAT. A significant benefit of non-surgical treatments was reported for pain scores. Although most of the observed differences between the two treatment groups were small, the improvements were at levels that may be of clinical importance. The review also highlights the wide range of non-surgical treatments being used for IAT. This evidence supporting the application of non-surgical therapy for participants with IAT must be balanced by the low methodological quality, small size, and short-term evidence currently available.

As the current NMA is the first to examine the comparative effectiveness of non-surgical treatments for IAT, it is challenging to directly compare the findings of the present study with those of NMAs. In this review, exercise as a stand-alone treatment was not found to consistently confer beneficial effects in reducing pain in participants with IAT in the short term. Still, the eccentric exercise was most effective in combination with soft tissue therapy.

Based on the results from NMA, the combination of eccentric exercise and soft tissue therapy was shown to be the most effective treatment to improve pain outcomes. In this study, 16 subjects were randomly assigned to either a soft tissue treatment and eccentric exercise group or an eccentric exercise-only group. The intervention was completed over 12 weeks, with outcomes assessed at baseline, 4, 8, 12, 26, and 52 weeks. Outcomes included the Victorian Institute of Sport Assessment Achilles-Specific Questionnaire (VISA-A), the numeric pain rating scale (NPRS), and the global rating of change (GROC). In addition, it was reported that soft tissue therapy plus eccentric exercise was more effective than eccentric exercise at improving function during short and long-term follow-up periods. However, the overall certainly of evidence was low in all included. No recommendation on the non-surgical intervention could be made from this review.

### Strengths and limitations of this review

This systematic review and network meta-analysis had various strengths and limitations. Two reviewers performed a comprehensive search and screening following standard guidelines for conducting systematic reviews (PRISMA) [39]. In addition, the network meta-analysis compared non-operative treatment options directly and indirectly. However, because of a miscellany of treatments, outcome measures and a lack of details, it wasn’t easy to analyse the designs of non-operative treatments.

Moreover, the main limitation of this systematic review was the low quality and overall certainty of evidence of included literature. On one hand, the characteristics of participants from the included studies were mainly sedentary women. It differed from the participants who commonly suffered from IAT, which were middle-aged, male, recreational athletes. As such, the results should be applied cautiously to participants with IAT. On the other hand, the diagnostic procedure was not explained in most of the included studies. The choice of diagnostic imaging and clinical examination may affect the diagnosis of IAT. In addition, most participants and assessors were not blinded to treatments applied in the RCT. The comorbidities of the participants were not listed in some trials. The timeframe for measuring objective outcomes varied across included studies. Since this review only included RCTs reporting VAS scores. It limited the variety of treatments for IAT evaluated in existing research. The treatment duration and follow-up time points applied to participants varied across included studies.

### Future research

This review highlighted that a wide variety of non-surgical interventions were being used to treat IAT. Current studies investigated the conventional treatments commonly used in rehabilitation, such as shockwave therapy, laser therapy and eccentric exercise. However, the overall effects were not significant among all included studies and the quality of evidence was low. None of the non-surgical treatment was superior to the other. Therefore, future research should investigate the clinical effects of specific novel non-surgical treatments in the long term.

## Conclusions

This is the first NMA of non-surgical treatment focusing on short-term pain control for IAT which eccentric exercise plus soft-tissue therapy was found to be the most effective treatment combination. However, the overall confidence in non-surgical treatments from all included trials was low. No recommendation of the best treatment option can be made from this review. Higher-quality trials on novel therapies with extended follow-up periods are needed to determine the most effective treatment modality.

## Data Availability

The datasets used and analysed during the current study are available from the corresponding author upon reasonable request.
